# Liberal versus restrictive red blood cell transfusion strategy in acute coronary syndrome and anemia: an updated systematic review and meta-analysis

**DOI:** 10.3389/fcvm.2025.1457400

**Published:** 2025-04-24

**Authors:** Sinda Hidri, Wajeeh Ur Rehman, Karam Gardezi, Jassim Zaheen Shah, Sai Venkata Siddhartha Masetti, Naiela E. Almansouri, Arslan Maan, Tirth Dave, Sumeja Catic, Simranjeet Singh Nagoke, Mohammad Ebad Ur Rehman, Huzaifa Ahmad Cheema, Adeel Ahmad, Raheel Ahmed, Abdelhamid Ben Selma, Mouhamed Amr Sabouni, Nabil Braiteh, Alon Yarkoni, Keyoor Patel, Afzal Ur Rehman

**Affiliations:** ^1^ Department of Internal Medicine, East Carolina University, Greenville, NC, United States; ^2^ Department of Internal Medicine, United Health Services Hospital, Johnson City, NY, United States; ^3^ Department of Internal Medicine, T.J. Samson Community Hospital, Glasgow, KY, United States; ^4^ Department of Cardiology, Hamad Medical Corporation, Doha, Qatar; ^5^ Department of Medicine, Kingston Public Hospital, Kingston, Jamaica; ^6^ Libyan Biotechnology Research Center, Tripoli, Libya; ^7^ Department of Internal Medicine, St Luke’s Hospital, Cedar Rapids, IA, United States; ^8^ Department of Medicine, Bukovinian State Medical University, Chernivtsi, Ukraine; ^9^ Department of Medicine, University of Zenica Faculty of Medicine, Zenica, Bosnia and Herzegovina; ^10^ Department of Medicine, Government Medical College and Hospital, Jammu, India; ^11^ Department of Medicine, Rawalpindi Medical University, Rawalpindi, Pakistan; ^12^ Department of Cardiology, King Edward Medical University, Lahore, Pakistan; ^13^ Department of Cardiovascular Medicine, Mayo Clinic, Rochester, MN, United States; ^14^ National Heart & Lung Institute, Imperial College London, London, United Kingdom; ^15^ Department of Cardiology, Royal Brompton Hospital, London, United Kingdom; ^16^ Department of Cardiovascular Disease, University of Alabama at Birmingham, Birmingham, AL, United States; ^17^ Department of Cardiology, Mercy One Siouxland Heart and Vascular Center, Sioux City, IA, United States; ^18^ UHS Heart & Vascular Institute, United Health Services Hospital, Johnson City, NY, United States

**Keywords:** ACS, transfusion, restrictive transfusion, liberal transfusion, acute coronary syndrome

## Abstract

**Background:**

It is uncertain whether a liberal red blood cell (RBC) transfusion strategy is superior to a restrictive approach in patients with acute coronary syndrome (ACS) and anemia.

**Methods:**

We searched MEDLINE, Embase, the Cochrane Library, and ClinicalTrials.gov from inception to April 2024 for randomized controlled trials (RCTs) comparing liberal and restrictive transfusion strategies in ACS patients with concurrent anemia.

**Results:**

Five RCTs (4,510 patients) were included in this meta-analysis. There was no significant difference between the liberal and restrictive RBC transfusion strategy groups in the risk of major adverse cardiovascular events (MACE) (RR 0.91, 95% CI: 0.68–1.21; *I*^2^ = 63%) and all-cause mortality (RR 0.85, 95% CI: 0.72, 1.00; *I*^2^ = 0%). A liberal transfusion strategy reduced the risk of myocardial infarction (MI) (RR 0.80, 95% CI: 0.66, 0.98; *I*^2^ = 0%). There were no significant differences between the two strategies in the risk of revascularization, heart failure, stroke, cardiac mortality, acute kidney injury or failure, and pneumonia, bacteremia, or infection. Liberal transfusion increased the risk of acute lung injury (RR 8.97, 95% CI: 1.65, 48.65; *I*^2^ = 0%).

**Conclusions:**

Our meta-analysis demonstrated that a liberal RBC transfusion strategy reduced the risk of MI and increased the risk of acute lung injury but did not affect other clinical outcomes compared to a restrictive approach in patients with mainly acute MI and anemia. New large-scale multicenter RCTs are required to confirm or refute our findings and provide more reliable results.

**Systematic Review Registration:**

PROSPERO (CRD42024506844).

## Introduction

The prevalence of anemia in patients with acute coronary syndromes (ACS) is reported to be 28% and is independently associated with a 50% increased risk of mortality in both men and women (1). Patients with ACS and anemia are older than those without anemia, have a higher prevalence of comorbidities, and are less likely to receive reperfusion therapy with either thrombolysis or primary percutaneous intervention (2).

In particular, anemia is an overlooked comorbidity in patients undergoing acute myocardial infarction (AMI), with 15%–43% having concomitant anemia in varying stages (3). Fundamentally, anemia results in diminished carrying capacity of oxygen in the blood, further compounding a preexisting supply/demand mismatch in cardiac tissue due to decreased coronary perfusion. Anemia has also been implicated in blunting the cytokine-mediated immune response generated by AMI, thereby limiting vascular healing capacity and thus worsening prognosis beyond the acute phase (4). Standardized treatment for AMI, such as heparin in the immediate period and antiplatelet agents that may be continued long-term, often potentiate the severity of anemia. Consequently, anemia has been associated with increased mortality in patients with AMI (3).

Augmenting the supply of oxygen delivered to infarcted tissue is the principle behind blood transfusion as therapy in individuals with ACS and anemia. However, transfusions are not necessarily benign interventions. Transfusion reactions, circulatory overload, acute lung injury, and infections, among others, are well-documented side effects of blood transfusions (5). Achieving the optimal balance between providing adequate oxygenation to compromised tissue and limiting the costs of transfusions has been a longstanding subject of debate.

There have been multiple attempts to compare outcomes between restrictive transfusion and liberal approaches. Historically, a liberal approach [hemoglobin (Hb) goal >10 g/dl] was favored. However, in 2014, the American Heart Association (AHA) and the American College of Cardiology (ACC) adopted guidelines against transfusing patients beyond Hb >8 g/dl in hemodynamically stable patients with AMI (6). Soon afterward, the American Association of Blood Banks (AABB) weighed in, citing no recommendation for or against a restrictive approach (7, 8). As per the 2023 European Society of Cardiology (ESC) guidelines for the management of ACS, no recommendation as to the optimal transfusion strategy can be made at present (9).

A previous meta-analysis by Abdelazeem et al. on transfusion strategies in patients with ACS and anemia, which included 821 patients, showed that a restrictive blood transfusion strategy was not associated with reduced all-cause mortality, recurrent MI, and heart failure exacerbation compared to a liberal strategy (10). The largest randomized controlled trial (RCT) on this topic to date has recently been published, including 3,500 adult patients (11). Therefore, we conducted this meta-analysis to integrate data from newer studies and compare the restrictive vs. liberal red blood cell (RBC) transfusion strategies in ACS patients with greater certainty and statistical power.

## Methods

The protocol of this review was registered with PROSPERO (CRD42024506844). The guidelines presented in the Cochrane Handbook for Systematic Reviews of Intervention (12) and the Preferred Reporting Items for Systematic Reviews and Meta-Analysis (PRISMA) statement (13) were followed for conducting this meta-analysis.

### Data sources and searches

We conducted electronic searches of the Cochrane Central Register of Controlled Trials (CENTRAL), MEDLINE (via Ovid), Embase (via Ovid), and ClinicalTrials.gov from inception to April 2024, with no language restrictions, using terms related to “acute coronary syndrome,” “myocardial infarction,” and “blood transfusion.” The detailed search strategies for each database are provided in Supplementary Table S1. Additionally, we reviewed reference lists of included studies and relevant systematic reviews and performed forward citation searching using Web of Science to identify further eligible articles.

### Eligibility criteria

We included studies that fulfilled the following PICOS criteria: (1) population: patients diagnosed with ACS and concurrent anemia; (2) intervention: a liberal RBC transfusion strategy; (3) comparator: a restrictive transfusion strategy; (4) outcomes: including any outcome of interest as defined below; and (5) study type: RCTs. ACS was defined as ST-segment elevation MI (STEMI), non-STEMI (NSTEMI), or unstable angina. Anemia was defined as a Hb level <10 g/dl or hematocrit ≤30%. We excluded case reports, single-arm studies, reviews, and animal studies.

### Study selection and data extraction

All literature retrieved from our searches was imported into Mendeley Desktop 1.19.8, and duplicates were removed. The remaining records were then uploaded to Rayyan, where two reviewers independently screened the titles and abstracts, followed by full-text screening. Any disagreements between the reviewers were resolved through discussion.

Two reviewers independently extracted data into a structured Excel spreadsheet, capturing study characteristics, participant characteristics, intervention and comparator details, and outcome data. In case of missing data, we planned to contact the study authors to ask for additional information; however, no such instance arose.

### Outcomes

The primary outcomes were major adverse cardiovascular events (MACE) defined as the composite of death, myocardial infarction (MI), and revascularization or congestive heart failure, and all-cause mortality. The secondary outcomes included the risk of MI, revascularization, heart failure, stroke, cardiac mortality, acute lung injury, acute kidney injury or failure, and the incidence of pneumonia, bacteremia, or infection.

### Quality and certainty of evidence assessment

The risk of bias assessment was conducted using the revised Cochrane “Risk of Bias” tool (RoB 2.0) for RCTs (14). The results were presented as a figure.

The quality of evidence for our primary outcomes was graded as very low, low, moderate, or high using the Grades of Recommendation, Assessment, Development, and Evaluation (GRADE) assessment tool. GRADE rates the quality of evidence based on the risk of bias, imprecision, inconsistency, and indirectness (15).

### Data analysis

Meta-analyses were conducted using Review Manager (RevMan) 5.4, employing a random-effects model with the DerSimonian-Laird variance estimator. The risk ratio (RR) was used as the effect measure. Heterogeneity was assessed using *χ*² test and *I*² statistic. We interpreted I^2^ values according to the guidance presented in the Cochrane Handbook for Systematic Reviews of Interventions (12). We conducted a sensitivity analysis on primary outcomes by excluding the trial by Carson et al. (16) because it was the only study that included patients with unstable angina. We also conducted sensitivity analyses by only including trials with a 30-day follow-up and trials comparing thresholds of Hb <10 g/dl and Hb <8 g/dl. Publication bias could not be assessed as there were less than 10 studies in the review.

## Results

### Study selection and characteristics

**Figure 1:**
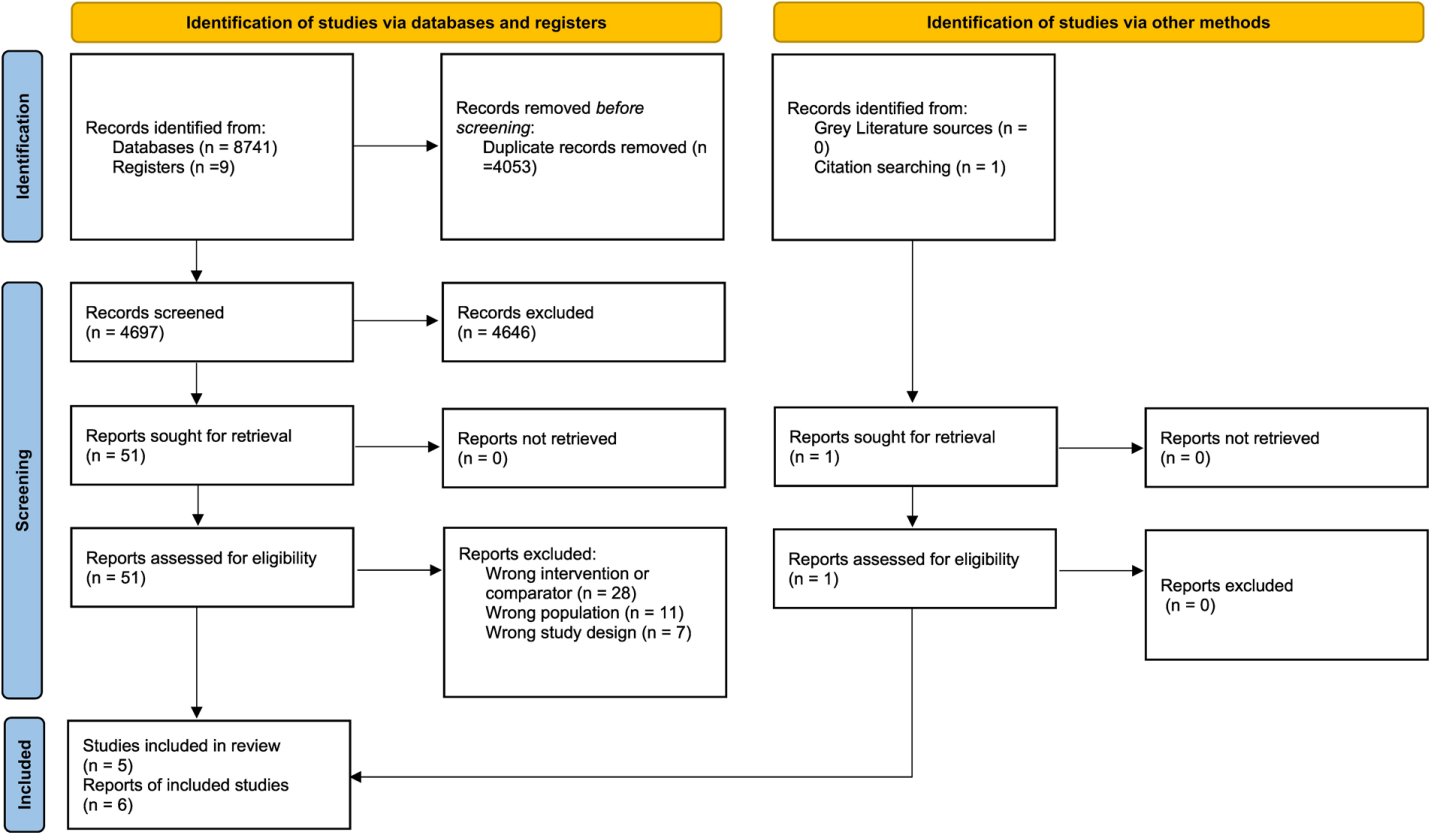
PRISMA flowchart of the study selection procedure.

We included a total of six reports from five RCTs in our meta-analysis (11, 16–20). The PRISMA flowchart depicts the detailed screening process (Figure 1). Table 1 illustrates the detailed characteristics of the studies. Most studies included STEMI and NSTEMI patients. The studies took place in a diverse range of countries. The follow-up period ranged from 30 days to 6 months.

**Table 1:** Characteristics of included studies.

Study ID	Location	Study design	No. of patients	Types of ACS	Liberal strategy (transfusion threshold)	Restrictive strategy (transfusion threshold)	Age (years)	Male (%)	Baseline Hb (g/dl)/Hct (%)	RBC transfusion, no. (%)	Number of RBC units transfused	Follow-up duration (month)
Carson et al. (16)	USA	RCT	110 (55 vs. 55)	STEMI, NSTEMI, unstable angina	Hb <10 g/dl	Hb <8 g/dl	67.3 (13.6) vs. 74.3 (11.1)	50.9 vs. 49.1	9.18 ± 0.64 vs. 8.97 ± 0.73	NR	87 vs. 27 (total)	30 days, 6 months
Carson et al. (11)	USA, Canada, Brazil, France, New Zealand, Australia	RCT	3,504 (1,755 vs. 1,749)	STEMI, NSTEMI	Hb <10 g/dl	Hb <8 g/dl	72.1 ± 11.6 vs. 72.2 ± 11.5	53.3 vs. 55.7	8.6 ± 0.8 vs. 8.6 ± 0.8	36.4 vs. 34.2	2.3 ± 2.1 vs. 2.4 ± 2.3	30 days
Cooper et al. (17)	USA	RCT	45 (21 vs. 24)	STEMI, NSTEMI	HCT <30%	HCT <24%	76.4 ± 13.5 vs. 70.3 ± 14.3	48 vs 54	26.9 ± 1.9 vs. 27.5 ± 2.4	100 vs. 54	2.5 ± 1.3 vs. 1.6 ± 2	30 days
Ducrocq et al. (18)	France and Spain	RCT	668 (324 vs. 342)	STEMI, NSTEMI	Hb ≤10 g/dl	Hb ≤8 g/dl	76 (69–84) vs 78 (69–85)	56.8 vs. 58.8	9.1 vs. 9.0	99.7 vs. 35.7	NR	30 days
Mistry et al. (20)	Canada, USA, Australia, New Zealand, China, Malaysia, Singapore, India, Denmark, Germany, Switzerland, Spain, Greece, Romania, Egypt, Israel, Brazil, Colombia, South Africa	RCT	194 (105 vs. 89)	STEMI, NSTEMI	Hb <8.5 g/dl (ward) and Hb <9.5 g/dl (ICU and OR)	Hb <7.5 g/dl	67.5 ± 8.9 vs. 67.4 ± 9.8	81 vs. 77.5	45.7 vs. 51.7	79 vs. 52.8	3.61 ± 4.61 vs. 1.82 ± 3.56	6 months

### Risk of bias in included studies

**Figure 2:**
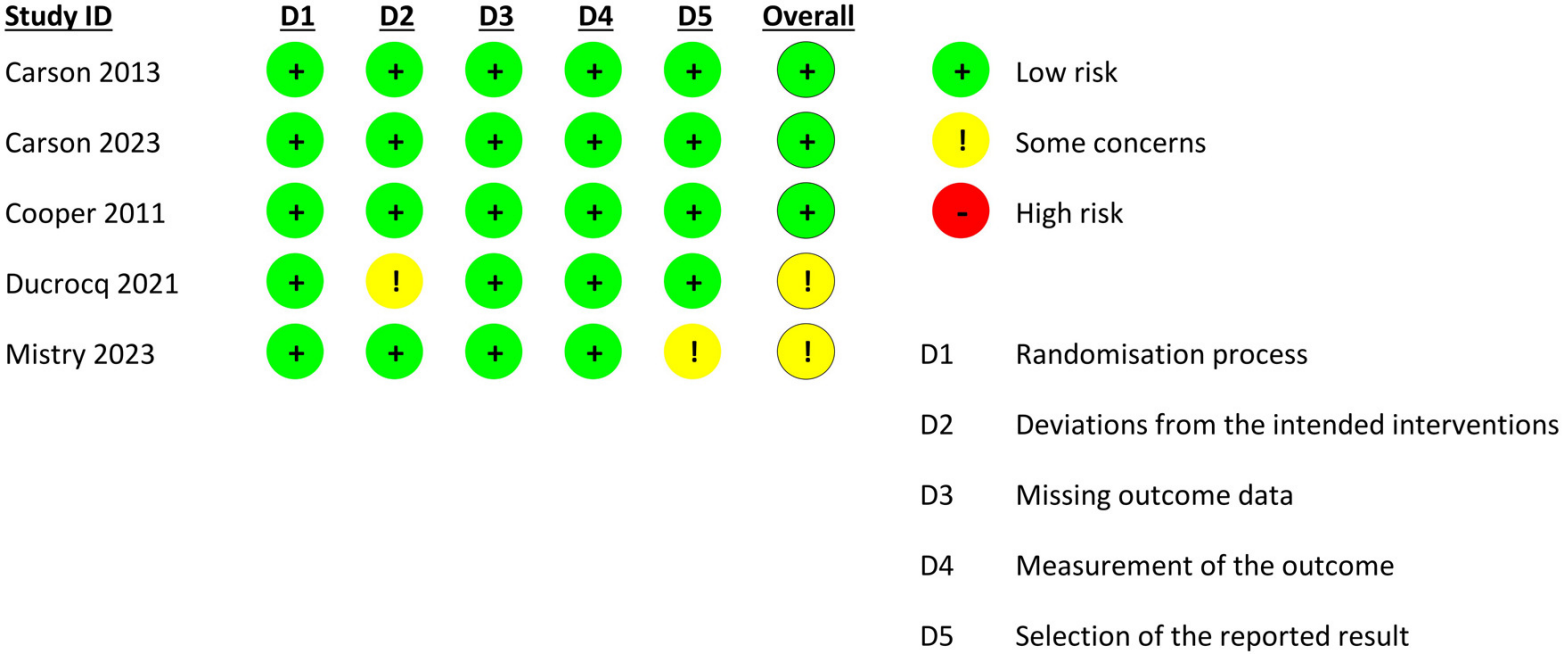
Risk of bias in included trials.

Three of the five included RCTs had a low risk of bias (Figure 2) (11, 16, 17). One trial had some concerns of bias in the deviations from the intended interventions domain (18), and one reported some concerns of bias in the selection of the reported results (20).

### Results of the meta-analysis

#### Primary outcomes

##### Major adverse cardiovascular events (MACE)

**Figure 3:**
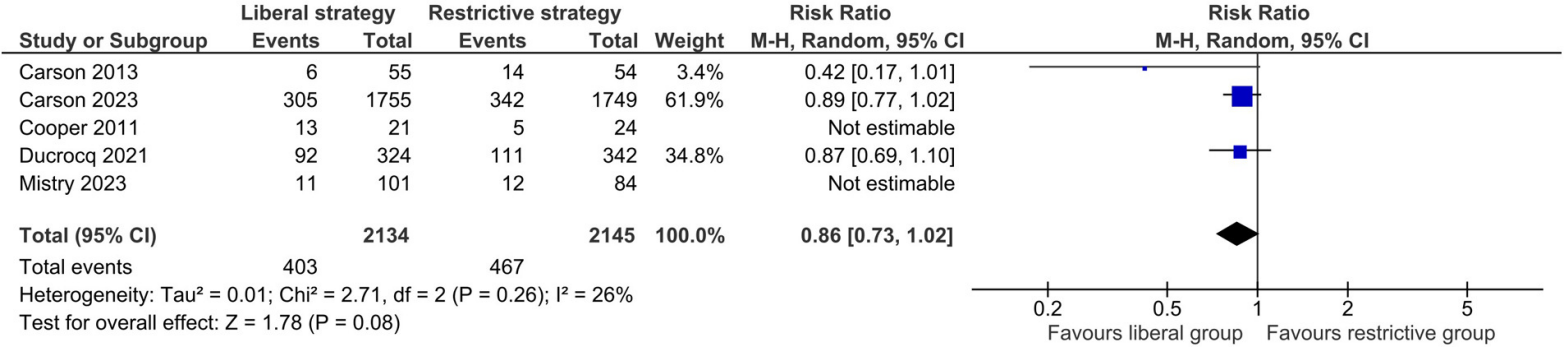
Effect of a liberal versus restrictive transfusion strategy on major adverse cardiovascular events.

No statistically significant difference was found between the liberal RBC transfusion and restrictive RBC transfusion groups regarding MACE (RR 0.91, 95% CI: 0.68–1.21; Figure 3). A substantial level of heterogeneity was reported among the studies for this outcome (*I*^2^ = 63%). The certainty of evidence was rated as moderate due to concerns about inconsistency (Supplementary Table S2). The results of the sensitivity analysis by the exclusion of Carson et al. (16) were similar to the primary analysis. Analyzing studies with a 30-day follow-up only also yielded similar results. However, only including studies comparing thresholds of Hb <10 g/dl and Hb <8 g/dl tilted the results in favor of the liberal strategy but remained nonsignificant (RR 0.86, 95% CI: 0.73–1.02; *I^2^* = 26%).

##### All-cause mortality

**Figure 4:**
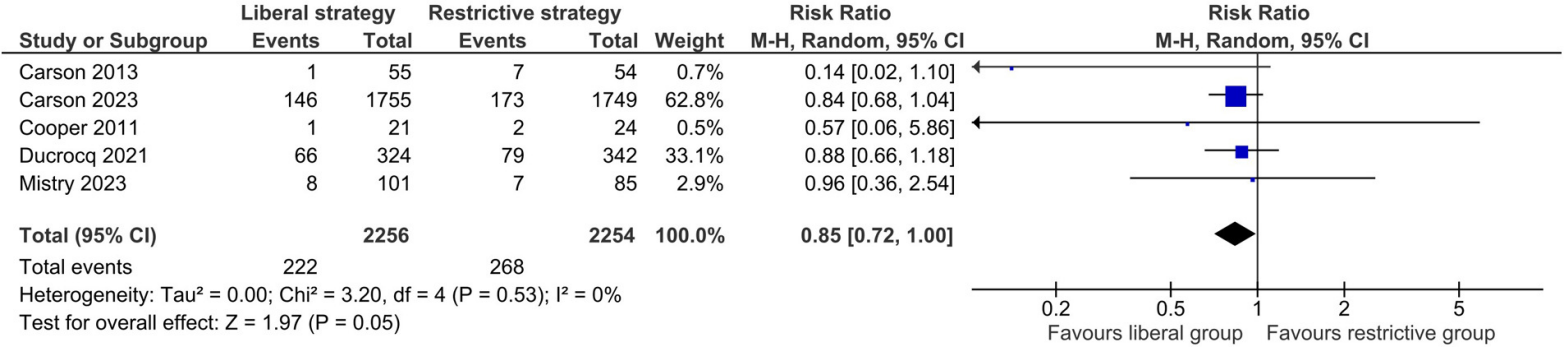
Effect of a liberal versus restrictive transfusion strategy on all-cause mortality.

There was no significant difference between the two transfusion strategies in the risk of all-cause mortality (RR 0.85, 95% CI: 0.72–1.00; *I*^2^ = 0%, Figure 4). The certainty of evidence was rated as moderate due to concerns about imprecision (Supplementary Table S2). The sensitivity analyses did not change the results substantially.

#### Secondary outcomes

A liberal RBC transfusion strategy reduced the incidence of MI (RR 0.80, 95% CI: 0.66–0.98; *I*^2^ = 0%; Supplementary Figure S1). There were no significant differences between the two groups in the risk of revascularization (RR 0.93, 95% CI: 0.63–1.38; *I*^2^ = 0%; Supplementary Figure S2), heart failure (RR 1.08, 95% CI: 0.62–1.89; *I*^2^ = 44%; Supplementary Figure S3), stroke (RR 0.90, 95% CI: 0.55–1.48; *I*^2^ = 0%; Supplementary Figure S4), and cardiac mortality (RR 0.95, 95% CI: 0.33–2.75; *I*^2^ = 88%; Supplementary Figure S5). Liberal blood transfusion increased the risk of acute lung injury (RR 8.97, 95% CI: 1.65–48.65; *I*^2^ = 0%; Supplementary Figure S6). The rates of acute kidney injury or failure (RR 0.95, 95% CI: 0.76–1.18; *I*^2^ = 18%; Supplementary Figure S7) and pneumonia, bacteremia, or infection (RR 1.15, 95% CI: 0.23–5.80; *I*^2^ = 50%; Supplementary Figure S8) were similar between the two transfusion strategies.

## Discussion

Our meta-analysis demonstrated that a liberal RBC transfusion strategy and a restrictive one in patients with mainly AMI did not differ in terms of the risk of MACE and all-cause mortality. Moreover, the two transfusion strategies did not differ in terms of the risk of revascularization, heart failure, stroke, cardiac mortality, acute kidney injury or failure, pneumonia, bacteremia, or infection. However, liberal blood transfusion reduced the risk of MI and increased the risk of acute lung injury.

As highlighted in our meta-analysis, the absence of difference in mortality between both transfusion groups aligns with findings from previous studies (21–23). A recent meta-analysis of RCTs by Abdelazeem et al. reported similar all-cause mortality, recurrent MI, revascularization, and heart failure exacerbation between the two groups; however, the included RCTs had a limited number of patients, with the cumulative sample size of the meta-analysis being only 821 patients (10). Our study included the MINT trial, the most recent RCT with the largest sample size (*N* = 3,500) to date, and hence, our analyses accumulate greater statistical power and provide more reliable results (11).

The two largest trials on this topic were incorporated into our study: the MINT and REALITY trials. REALITY was a non-inferiority trial; the study was underpowered regarding superiority (18). The non-inferiority of the restrictive transfusion observed at 30 days was not found at one year: the restrictive strategy was associated with more frequent MACE than the liberal strategy at one year. However, these findings are the results of a *post hoc* analysis and should be considered exploratory (19).

A study conducted by Wang et al. showed no difference in risk for MI between the two transfusion strategies (24). Their study is in agreement with previous studies that suggest that the liberal strategy is associated with more harm than benefit (25–27). However, in our study, liberal transfusion led to a reduction in the risk of MI. Patients with acute myocardial ischemia are subject to bleeding and acute anemia, especially when they are using anti-platelet medications or therapeutic anticoagulants, and transfusion might be warranted to enhance oxygen delivery and mitigate bleeding risks (28–30). Furthermore, it is to be noted that we found a trend toward reduced all-cause mortality with a liberal transfusion strategy. While these findings favor liberal RBC transfusion, the clinical benefit is uncertain. In addition, the increased risk of acute lung injury seen in our analyses needs to be considered, although the extremely wide 95% CIs suggest that it might be a spurious finding. Moreover, existing literature has shown that RBC transfusion is associated with acute kidney injury (31); however, our analysis did not corroborate this. Insufficient RCTs compound the controversy surrounding these findings to provide conclusive evidence. Large prospective trials are needed in the future to assess the outcomes of both strategies.

One final aspect to consider when choosing the liberal transfusion strategy is the cost burden and the financial implication, especially in the USA, a country where transfusion shortage is a concern (32). The REALITY trial compared the incremental cost-effectiveness ratio at 30 days and the 1-year quality-adjusted life year (QALY) between both strategies, and results suggest that the restrictive option is more cost-effective than liberal transfusion (33). Transfusion remains a costly treatment, and blood transfusions come with an increased risk of transfusion reactions and use of medical resources (18). Thus, this should be taken into account when making any recommendations.

This review had several limitations. Many of the RCTs included were open-label trials and carried a risk of performance bias, as blinding patients and healthcare providers was not possible. Also, some RCTs, such as the MINT trial, lacked central adjudication for many of the included outcomes. Furthermore, there was some variation in the length of follow-up and the transfusion thresholds between the studies, which could have led to heterogeneity in our results. Additionally, as this is a study-level analysis, and we did not have access to individual patient data, we were unable to investigate further subgroups of interest, such as STEMI vs. NSTEMI patients. Finally, we could not assess publication bias as our meta-analysis included less than 10 studies.

## Conclusion

Our meta-analysis showed no statistically significant difference in the risk of MACE and all-cause mortality between the liberal and restrictive transfusion groups in patients with ACS, mainly AMI. Among secondary outcomes, there was a lower risk of MI and an elevated incidence of acute lung injury in the liberal transfusion group. There was no statistically significant difference between the two transfusion groups in the risk of heart failure, stroke, cardiac mortality, acute kidney injury or failure, pneumonia, bacteremia or infection, and the need for revascularization. Further, large-scale RCTs are required to establish which of these two transfusion strategies is better.

## Data Availability

The original contributions presented in the study are included in the article/Supplementary Material, further inquiries can be directed to the corresponding authors.
