# Supplementary material for: Liberal versus restrictive red blood cell transfusion strategy in acute coronary syndrome and anemia: an updated systematic review and meta-analysis
Source: Front Cardiovasc Med. 2025 Apr 24;12:1457400. doi: 10.3389/fcvm.2025.1457400 (PMC12060722; doi:10.3389/fcvm.2025.1457400)

Supplementary Table 1. Search strategy for databases.

MEDLINE (Ovid)

1. Blood Transfusion/
2. Blood Component Transfusion/
3. Erythrocyte Transfusion/
4. ((erythrocyte* or red cell* or blood or RBC*) adj5 (transfus* or unit*)).tw,kf.
5. ((red cell* or RBC* or erythrocyte* or red blood cell* or whole blood or transfus*) adj5 (trigger* or level* or threshold* or rule* or target* or restrict* or liberal* or reduc* or limit*)).tw,kf.
6. (allogeneic blood or (unit* adj2 blood) or allogenic blood or (blood adj2 exposure) or donor blood or blood product* or blood component* or blood support).ti,ab.
7. (h?emotransfus* or hypertransfus* or h?emotherap*).tw,kf.
8. (red cell* or erythrocyte* or transfus* or whole blood or RBC*).ti.
9. Blood Component Transfusion/ not (Exchange Transfusion, Whole Blood/ or Plasma Exchange/ or Platelet Transfusion/ or Exp Leukocyte Transfusion/)
10. Erythrocytes/ or (red cell* or red blood cell* or erythrocyte* or RBC*).ti.
11. 9 and 10
12. or/1-8,11
13. Exp Myocardial Ischemia/
14. ((myocardial or myocardium or subendocardial or transmural or cardiac or cardial or coronary or heart) adj2 (infarct* or postinfarct* or hypoxi* or anoxi* or failure* or decompensation or insufficien*)).tw.
15. (heart disease* or coronary disease* or IHD or CIHD or CHD).tw.
16. (myocardial dysfunction or angina or stenocardia).tw.
17. ((ischemi* or ischaemi*) adj2 (myocardium or myocardial or heart or coronary or cardiac or cardial or subendocardial or cardiomyopath*)).tw.
18. ((artery occlusion* or artery disease* or arterioscleros* or atheroscleros*) adj2 coronary).tw.
19. or/13-18
20. Acute Coronary Syndrome/
21. Exp Myocardial Infarction/
22. Exp Coronary Thrombosis/
23. Coronary Thrombosis.tw.
24. Acute Coronary.tw.
25. Exp Angina, Unstable/
26. Myocardial Infarct*.tw.
27. Heart Infarct*.tw.
28. Acs.tw.
29. Ami.tw.

30. (Coronary Adj3 Syndrome*).tw.
31. Acute Angina.tw.
32. (Unstable Adj3 Angina).tw.
33. Unstable Coronary.tw.
34. or/20-33
35. 19 or 34
36. 12 and 35

CENTRAL (Cochrane Library)

#1 MeSH Descriptor: [Blood Transfusion] This Term Only
#2 MeSH Descriptor: [Blood Component Transfusion] Explode All Trees
#3 MeSH Descriptor: [Erythrocyte Transfusion] This Term Only
#4 (erythrocyte* or "red cell" or "red cells" or blood or RBC*) Near/3 (transfus* or unit*)
#5 (("red cell" or "red cells" or RBC* or erythrocyte* or "red blood cell" or "red blood cells" or "whole blood" or transfus*) Near/3 (trigger* or level* or threshold* or rule* or target* or restrict* or liberal* or reduc* or limit*))
#6 (("red cell" or "red cells" or blood) Near/3 (management or sparing or support or strateg*))
#7 ("allogeneic blood" or (unit* Near/2 blood) or "allogenic blood" or (blood Near/2 exposure) or "donor blood" or "blood product" or "blood products" or "blood component" or "blood components" or "blood support")
#8 (h*emotransfus* or hypertransfus* or h*emotherap*)
#9 ("red cell" or "red cells" or "red blood cell" or "red blood cells" or RBC* or transfus*):ti
#10 #1 Or #2 Or #3 Or #4 Or #5 Or #6 Or #7 Or #8 Or #9
#11 MeSH Descriptor: [Myocardial Ischemia] Explode All Trees
#12 ((myocardial or myocardium or subendocardial or transmural or cardiac or cardial or coronary or heart) Adj2 (infarct* or postinfarct* or hypoxi* or anoxi* or failure* or decompensation or insufficien*))
#13 (heart disease* or coronary disease* or IHD or CIHD or CHD)
#14 (myocardial dysfunction or angina or stenocardia)
#15 ((ischemi* or ischaemi*) Adj2 (myocardium or myocardial or heart or coronary or cardiac or cardial or subendocardial or cardiomyopath*))
#16 ((artery occlusion* or artery disease* or arterioscleros* or atheroscleros*) Adj2 coronary)
#17 #11 Or #12 Or #13 Or #14 Or #15 Or #16
#18 MeSH Descriptor: [Acute Coronary Syndrome] Explode All Trees
#19 MeSH Descriptor: [Myocardial Infarction] Explode All Trees
#20 MeSH Descriptor: [Coronary Thrombosis] Explode All Trees
#21 Coronary Thrombosis
#22 Acute Coronary
#23 MeSH Descriptor: [Angina, Unstable] Explode All Trees

#24 Myocardial Infarct*
#25 Heart Infarct*
#26 Acs
#27 Ami
#28 (Coronary Adj3 Syndrome*)
#29 Acute Angina
#30 (Unstable Adj3 Angina)
#31 Unstable Coronary
#32 #18 Or #19 Or #20 Or #21 Or #22 Or #23 Or #24 Or #25 Or #26 Or #27 Or #28 Or #29 Or #30 Or #31
#33 #17 Or #32
#35 #10 And #33

Embase (Ovid)

1. *Blood Transfusion/
2. Blood Component Therapy/
3. Erythrocyte Transfusion/
4. ((Erythrocyte* Or Red Blood Cell* Or Red Cell* Or Blood Or RBC*) Adj5 (Transfus* Or Unit*)).Tw,Kw.
5. ((Red Cell* Or RBC* Or Erythrocyte* Or Red Blood Cell* Or Whole Blood Or Transfus*) Adj3 (Trigger* Or Level* Or Threshold* Or Rule* Or Target* Or Restrict* Or Liberal* Or Reduc* Or Limit*)).Tw,Kw.
6. (Allogeneic Blood Or (Unit* Adj2 Blood) Or Allogenic Blood Or (Blood Adj2 Exposure) Or Donor Blood Or Blood Product* Or Blood Component* Or Blood Support).Tw,Kw.
7. (H?emotransfus* Or H?emotherap* Or Hypertransfus*).Tw,Kw.
8. (Transfus* Or Red Cell* Or Red Blood Cell* Or RBC* Or Whole Blood).Ti.
9. Or/1-8
10. Exp Heart Muscle Ischemia/
11. ((Myocardial Or Myocardium Or Subendocardial Or Transmural Or Cardiac Or Cardial Or Coronary Or Heart) Adj2 (Infarct* Or Postinfarct* Or Hypoxi* Or Anoxi* Or Failure* Or Decompensation Or Insufficien*)).Tw.
12. (Heart Disease* Or Coronary Disease* Or IHD Or CIHD Or CHD).Tw.
13. (Myocardial Dysfunction Or Angina Or Stenocardia).Tw.
14. ((Ischemi* Or Ischaemi*) Adj2 (Myocardium Or Myocardial Or Heart Or Coronary Or Cardiac Or Cardial Or Subendocardial Or Cardiomyopath*)).Tw.
15. ((Artery Occlusion* Or Artery Disease* Or Arterioscleros* Or Atheroscleros*) Adj2 Coronary).Tw.
16. Or/10-15
17. Exp Acute Coronary Syndrome/
18. Exp Heart Infarction/

19. Coronary Artery Thrombosis/
20. Coronary Thrombosis.Tw.
21. Acute Coronary.Tw.
22. Exp Unstable Angina Pectoris/
23. Myocardial Infarct*.Tw.
24. Heart Infarct*.Tw.
25. Acs.Tw.
26. Ami.Tw.
27. (Coronary Adj3 Syndrome*).Tw.
28. Acute Angina.Tw.
29. (Unstable Adj3 Angina).Tw.
30. Unstable Coronary.Tw.
31. Or/17-30
32. 16 or 31
33. 9 and 32

ClinicalTrials.gov

Condition: "acute coronary syndrome," "ischaemic heart disease," "stemi," "nstemi," or "unstable angina"

Interventions: "red cell transfusion," "blood transfusion," "red cells," "rbcs," or "red blood cells"

Supplementary Table 2. Grading of recommendations assessment, development, and evaluation (GRADE) summary of findings from randomized controlled trials.

**Outcome**	**No. of participants (studies)**	**Effect estimate (95% CI)**	**Risk of bias**	**Inconsistency**	**Indirectness**	**Imprecision**	**Quality of Evidence (GRADE)**
Major adverse cardiovascular events	4509 (5)	RR 0.91 (0.68, 1.21)	Not serious	Serious	Not serious	Not serious	⨁⨁⨁⊖<br>Moderate
All-cause mortality	4510 (5)	RR 0.85 (0.72, 1.00)	Not serious	Not serious	Not serious	Serious	⨁⨁⨁⊖<br>Moderate

Supplementary Figure 1. Effect of liberal vs. restrictive red blood cell transfusion on the risk of myocardial infarction.

	Liberal strategy		Restrictive strategy			Risk Ratio
Study or Subgroup	Events	Total	Events	Total	Weight	M-H, Random, 95% CI
Carson 2013	5	55	7	54	3.5%	0.70 [0.24, 2.07]
Carson 2023	126	1755	149	1749	78.8%	0.84 [0.67, 1.06]
Cooper 2011	0	21	1	24	0.4%	0.38 [0.02, 8.83]
Ducrocq 2021	21	324	33	342	14.8%	0.67 [0.40, 1.14]
Mistry 2023	4	95	5	79	2.5%	0.67 [0.18, 2.39]
**Total (95% CI)**		**2250**		**2248**	**100.0%**	**0.80 [0.66, 0.98]**
Total events	156		195			

Heterogeneity: Tau² = 0.00; Chi² = 0.98, df = 4 (P = 0.91); I² = 0%

Test for overall effect: Z = 2.13 (P = 0.03)

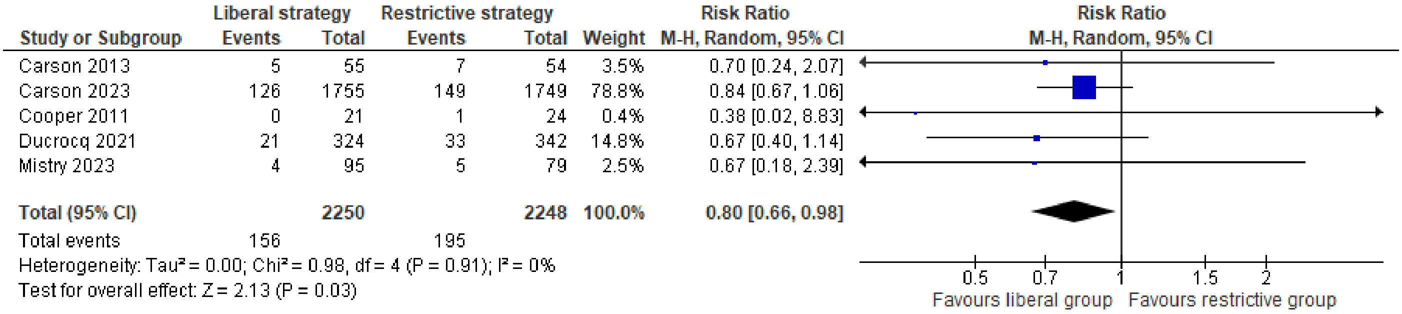

Supplementary Figure 2. Effect of liberal vs. restrictive red blood cell transfusion on the risk of revascularization.

	Liberal strategy		Restrictive strategy			Risk Ratio
Study or Subgroup	Events	Total	Events	Total	Weight	M-H, Random, 95% CI
Carson 2013	0	55	2	54	1.7%	0.20 [0.01, 4.00]
Carson 2023	39	1755	43	1749	85.4%	0.90 [0.59, 1.39]
Ducrocq 2021	6	324	5	342	11.3%	1.27 [0.39, 4.11]
Mistry 2023	1	95	0	77	1.5%	2.44 [0.10, 59.00]
**Total (95% CI)**		**2229**		**2222**	**100.0%**	**0.93 [0.63, 1.38]**
Total events	46		50			

Heterogeneity: $Tau^2 = 0.00$; $Chi^2 = 1.66$, $df = 3$ ($P = 0.65$); $I^2 = 0\%$
Test for overall effect: $Z = 0.37$ ($P = 0.71$)

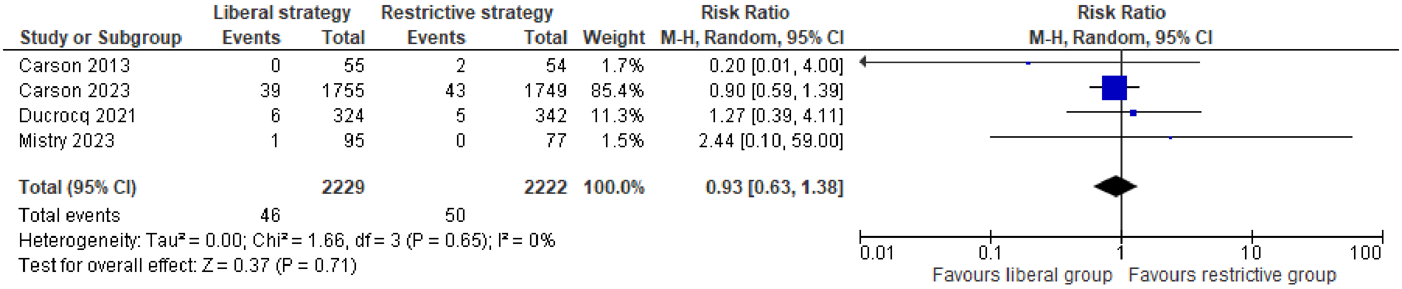

Supplementary Figure 3. Effect of liberal vs. restrictive red blood cell transfusion on the risk of heart failure.

Study or Subgroup	Liberal strategy Events	Liberal strategy Total	Restrictive strategy Events	Restrictive strategy Total	Weight	Risk Ratio M-H, Random, 95% CI
Carson 2013	2	55	7	54	10.9%	0.28 [0.06, 1.29]
Carson 2023	111	1755	102	1749	51.5%	1.08 [0.84, 1.41]
Cooper 2011	6	21	2	24	11.3%	3.43 [0.77, 15.20]
Ducrocq 2021	12	324	11	342	26.3%	1.15 [0.52, 2.57]
**Total (95% CI)**		**2155**		**2169**	**100.0%**	**1.08 [0.62, 1.89]**
Total events	131		122			

Heterogeneity: Tau² = 0.14; Chi² = 5.33, df = 3 (P = 0.15); I² = 44%
Test for overall effect: Z = 0.28 (P = 0.78)

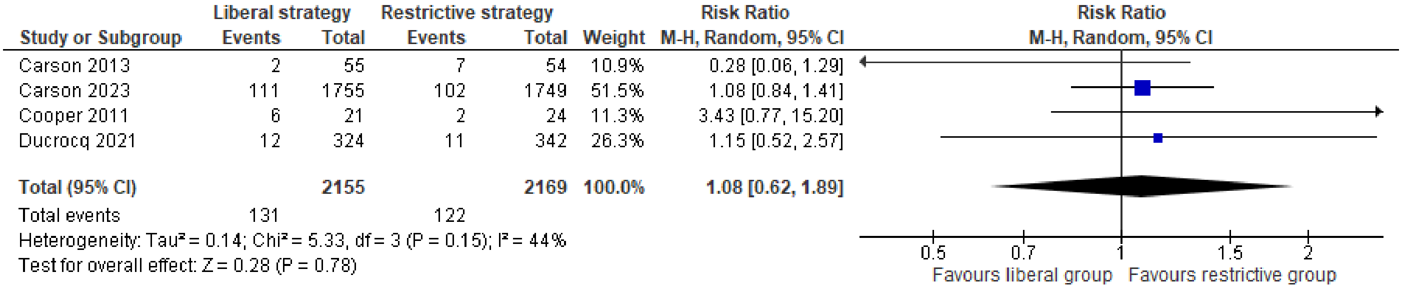

Supplementary Figure 4. Effect of liberal vs. restrictive red blood cell transfusion on the risk of stroke.

Study or Subgroup	Liberal strategy Events	Liberal strategy Total	Restrictive strategy Events	Restrictive strategy Total	Weight	Risk Ratio M-H, Random, 95% CI
Carson 2013	1	55	0	54	2.4%	2.95 [0.12, 70.77]
Carson 2023	26	1755	30	1749	91.1%	0.86 [0.51, 1.45]
Ducrocq 2021	2	324	2	342	6.5%	1.06 [0.15, 7.45]
**Total (95% CI)**		**2134**		**2145**	**100.0%**	**0.90 [0.55, 1.48]**
Total events	29		32			

Heterogeneity: Tau² = 0.00; Chi² = 0.58, df = 2 (P = 0.75); I² = 0%

Test for overall effect: Z = 0.41 (P = 0.68)

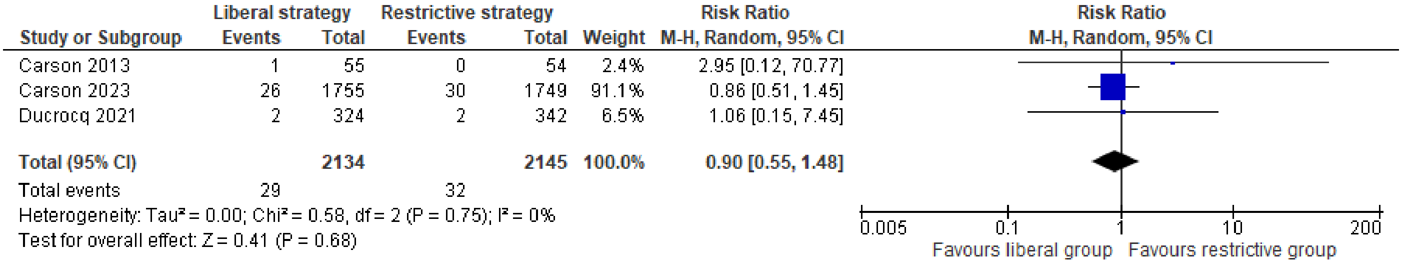

Supplementary Figure 5. Effect of liberal vs. restrictive red blood cell transfusion on the risk of cardiac mortality.

	Liberal strategy		Restrictive strategy			Risk Ratio
Study or Subgroup	Events	Total	Events	Total	Weight	M-H, Random, 95% CI
Carson 2023	56	1755	97	1749	53.9%	0.58 [0.42, 0.79]
Ducrocq 2021	21	324	13	342	46.1%	1.71 [0.87, 3.35]
**Total (95% CI)**		**2079**		**2091**	**100.0%**	**0.95 [0.33, 2.75]**
Total events	77		110			

Heterogeneity: Tau² = 0.52; Chi² = 8.11, df = 1 (P = 0.004); I² = 88%
Test for overall effect: Z = 0.10 (P = 0.92)

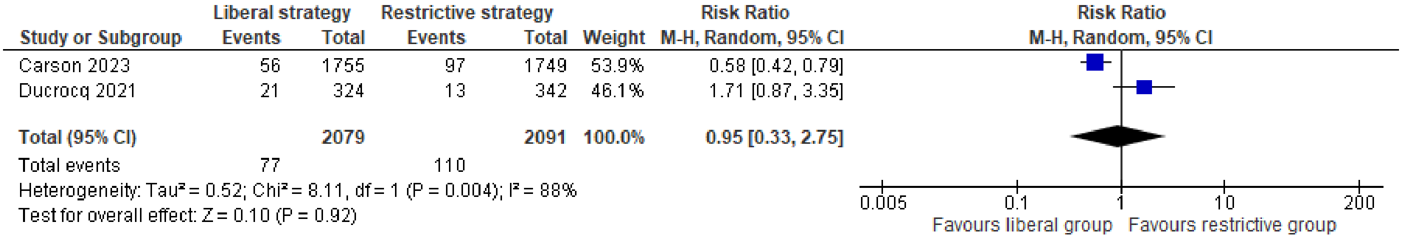

Supplementary Figure 6. Effect of liberal vs. restrictive red blood cell transfusion on the risk of acute lung injury.

	Liberal strategy		Restrictive strategy			Risk Ratio
Study or Subgroup	Events	Total	Events	Total	Weight	M-H, Random, 95% CI
Carson 2023	6	1754	0	1748	34.6%	12.96 [0.73, 229.80]
Ducrocq 2021	7	324	1	342	65.4%	7.39 [0.91, 59.72]
**Total (95% CI)**		**2078**		**2090**	**100.0%**	**8.97 [1.65, 48.65]**
Total events	13		1			

Heterogeneity: Tau² = 0.00; Chi² = 0.10, df = 1 (P = 0.75); I² = 0%
Test for overall effect: Z = 2.54 (P = 0.01)

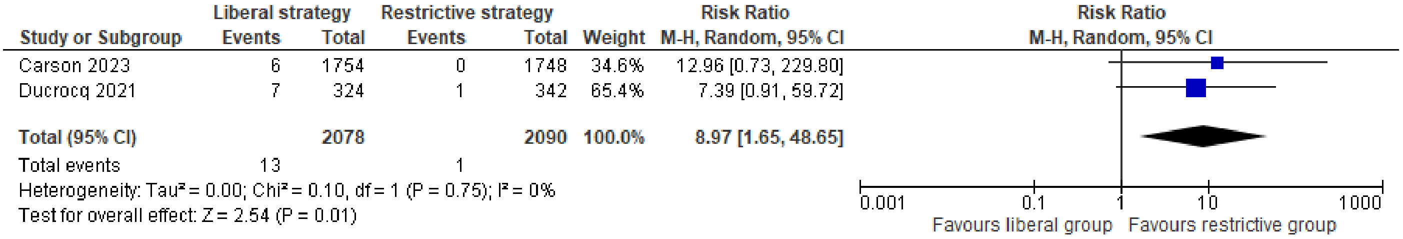

Supplementary Figure 7. Effect of liberal vs. restrictive red blood cell transfusion on the risk of acute kidney injury or failure.

Study or Subgroup	Liberal strategy Events	Liberal strategy Total	Restrictive strategy Events	Restrictive strategy Total	Weight	Risk Ratio M-H, Random, 95% CI
Carson 2023	233	1755	233	1749	82.7%	1.00 [0.84, 1.18]
Ducrocq 2021	23	324	33	342	17.3%	0.74 [0.44, 1.23]
**Total (95% CI)**		**2079**		**2091**	**100.0%**	**0.95 [0.76, 1.18]**
Total events	256		266			

Heterogeneity: Tau² = 0.01; Chi² = 1.23, df = 1 (P = 0.27); I² = 18%
Test for overall effect: Z = 0.49 (P = 0.63)

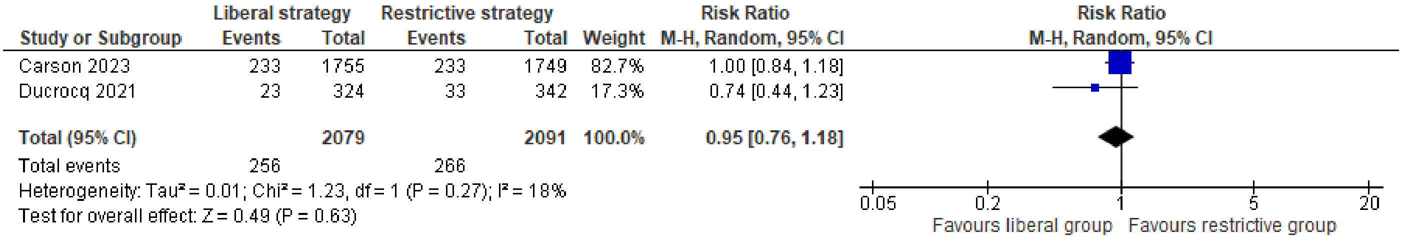

Supplementary Figure 8. Effect of liberal vs. restrictive red blood cell transfusion on the risk of pneumonia, bacteremia, or infection.

Study or Subgroup	Liberal strategy Events	Liberal strategy Total	Restrictive strategy Events	Restrictive strategy Total	Weight	Risk Ratio M-H, Random, 95% CI
Carson 2013	0	55	2	54	19.6%	0.20 [0.01, 4.00]
Carson 2023	153	1755	166	1749	59.8%	0.92 [0.74, 1.13]
Ducrocq 2021	5	324	0	342	20.7%	11.61 [0.64, 209.11]
**Total (95% CI)**		**2134**		**2145**	**100.0%**	**1.15 [0.23, 5.80]**
Total events	158		168			

Heterogeneity: Tau² = 1.13; Chi² = 3.98, df = 2 (P = 0.14); I² = 50%

Test for overall effect: Z = 0.17 (P = 0.87)

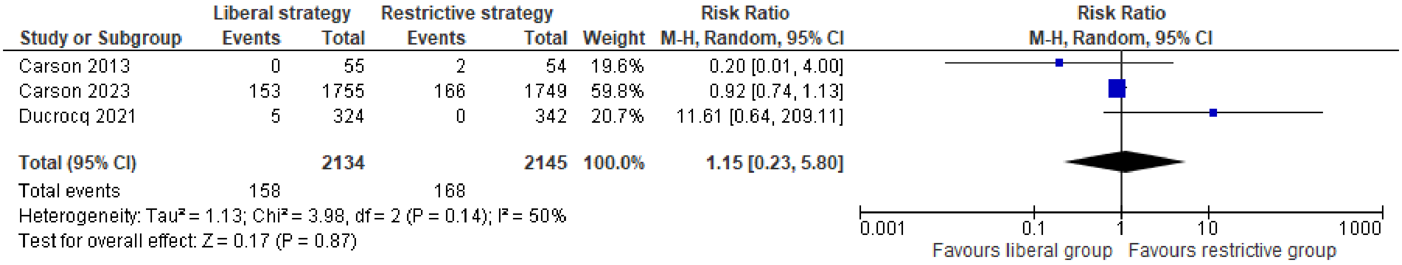

Supplement: Supplementary file 1 [file Datasheet1.pdf]
